# *The Production of Antibodies* (1941) by F. M. Burnet or by Burnet, Freeman, Jackson and Lush: Collaboration in research

**DOI:** 10.1177/09677720251395368

**Published:** 2025-11-18

**Authors:** Brian K Hall

**Affiliations:** 1 Department of Biology, 3688 Dalhousie University, Halifax, NS, Canada

**Keywords:** Frank Burnet, virology, immunology, production of antibodies, Mavis Freeman, Alan Vaugh Jackson, Dora Lush, Edward Derrick, Frank Fenner

## Abstract

A slim 75-page ‘book,’ *The Production of Antibodies: A Review and a Theoretical Discussion* was published 84 years ago, in 1941. The authorship is normally attributed to Francis MacFarlane Burnet (1889–1985), 1960 Noble Laureate for his research on acquired immune tolerance and acknowledged as the most famous Australian scientist. A revised edition in 1949 was co-authored with Frank Fenner (1914–2010), another distinguished Australian virologist, best remembered for the elimination of smallpox in Australia and for control of the rabbit population. The curiosity and the topic of this paper is that three collaborators are listed on the title page of the 1941 book – Mavis Freeman, A. V. (Alan Vaughan) Jackson and Dora Lush. All three worked with Burnet at the Walter and Eliza Hall Institute in Melbourne between 1936 and 1939/1940 during which time they were co-authors on 25 research papers. Who were these collaborators, what did they contribute to the book and why the confusion over authorship? This journey takes us into research on influenza, poliomyelitis, smallpox, myxomatosis, herpes, Q fever and scrub typhus undertaken by brilliant scientists who contributed to important advances in virology and immunology with one tragic consequence.

## Introduction

In 1941, Francis MacFarlane Burnet's slim, 75-page monograph, *The Production of Antibodies* was published. In 1949 a slightly larger 142-page revised edition was published with Frank Fenner as co-author.^1,2^ Australian virologist Frank John Fenner (1914–2010) is most well-known for his research and application leading to the eradication of smallpox in Australia and for control of the rabbit population with *Myxoma* virus. In the 1949 revised edition Burnet and Fenner first proposed the concept that antigens stimulate the production of antibodies by adapting a specific class of intracellular molecules. This, along with the concept of acquired immune tolerance, laid the foundation for modern immunology and adaptive immunity.^3,4^

The full title of the 1941 ‘book’ was *The Production of Antibodies. A Review and a Theoretical Discussion.*^1^ It seems a bit of a cheek to call it a book, given its size, although on my shelves I have a book of 50 pages on embryos, genes and evolution, two books of 50 and 80 pages on tissue culture, and two of 72 and 86 pages on photography and microscopy.^5–9^ So, small is OK.

**Figure 1.**
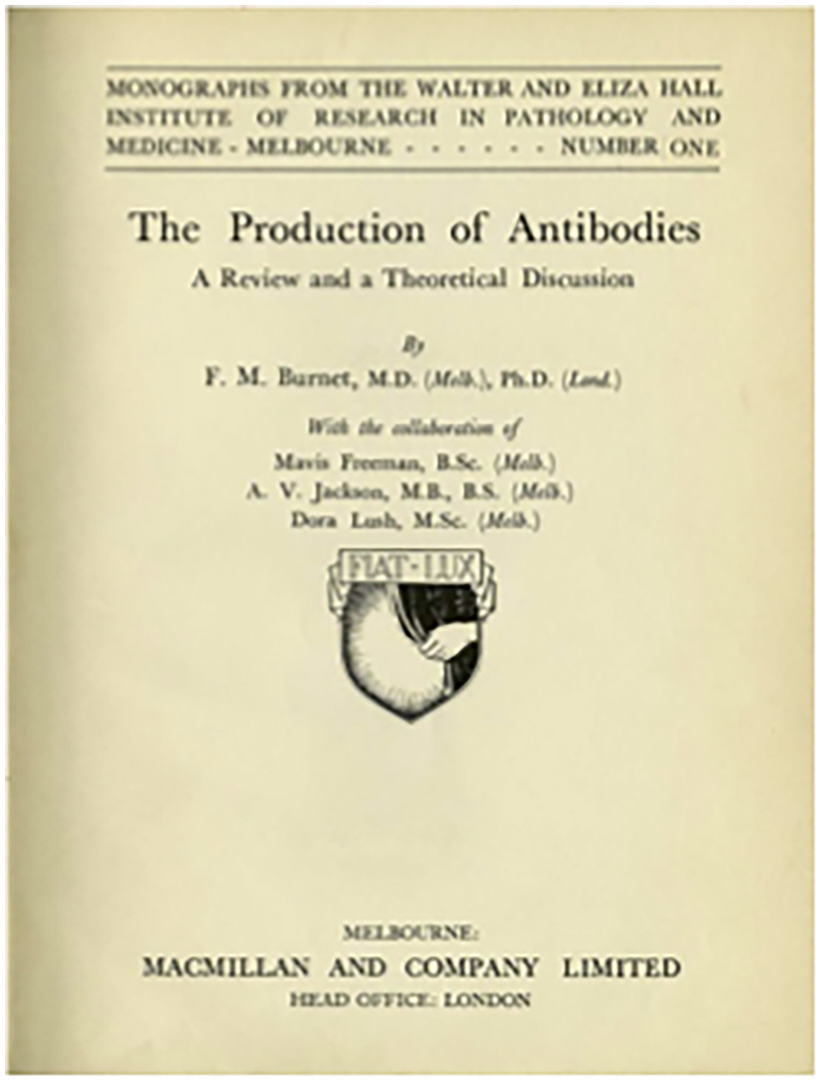
Title page of *The Production of Antibodies* (1941).

Furthermore, Burnet's ‘book’ was published as a Monograph of the Walter and Eliza Hall Institute (WEHI) of Research in Pathology and Medicine, Melbourne;^1^ hereafter the WEHI. So too, was the revised edition, both editions being identified as Monograph No. 1 (Figure 1). True it had a commercial publisher (MacMillan, Melbourne). Today it would be treated as part of a series, but times have changed (and no other monographs appeared in the ‘series’).

Fenner may have provided the reason for the length of the book and why it was published as a ‘book’. In his biographical memoir of Frank Macfarlane Burnet,^10^ Fenner tells us that ‘his [Burnet's] paper on these results and their implications [on the antibody response of rabbits to staphylococcal toxoid] was rejected by the British journal to which it had been sent, but this only stimulated him to collect further information on the production of antibodies and to publish it in an Institute monograph, *The Production of antibodies*…’ (p. 117). So, Burnet elected, essentially, to self-publish without peer review in a monograph of the Institute of which he was Deputy Director.

## Authorship/collaboration?

The curiosity that will occupy us here, is that, while, nowadays, the monograph is almost always cited as by F. M. Burnet, in fact, three collaborators are listed on the title page, complete with their degrees *–* Mavis Freeman, A. V. Jackson and Dora Lush – (Figure 1). The three overlapped as assistants at the WEHI between 1936 and 1939/1940. The original ‘authorship’ can be found in a Supplement to *Nature* (January 17, 1942, iii) advertising MacMillan's New Books. The ad reads: ‘*Production of Antibodies. A Review and a Theoretical Discussion F. M. BURNET, M.D., Ph.D.,* with the collaboration of *MAVIS FREEMAN, B.Sc., A. V. JACKSON, M.B., B.S., and DORA LUSH, M.Sc’.*

In a review in *Nature* of 23 May 1942 by the bacteriologist A. A. Miles, the book is described as: *The Production of Antibodies. A Review and a Theoretical Discussion*. By Dr F. M. Burnet, with the collaboration of Mavis Freeman, A. V. Jackson, Dora Lush. (Monographs from the Walter and Eliza Hall Institute of Research in Pathology and Medicine, Melbourne, No. 1.) pp. viii + 76. (Melbourne and London: Macmillan and Co., Ltd, 1941.) 8*s*. 6*d*. net.

As far as I can determine, the collaborators played no role in writing the monograph and there is no evidence that Burnet requested their comments on the manuscript. So, who were the three contributors and what was their role in the production of the monograph and in the research upon which it is based?

## The three collaborators

In his biographical memoir Fenner indicates that Burnet was very careful in selecting ‘graduate assistants’.^10^ Fenner lists 12 women as having been assistants between 1928 and 1963. No men are identified although one of the collaborators on the book was a man, *A. V. Jackson* (see below). Of the other two who collaborated on the monograph, *Mavis Freeman* was with Burnet for 12 years (1928 to 1940) and *Dora Lush* for five (1934–1939).^11^

As we will see, the degree of skill and collaboration between these three graduate assistants and with Burnet is amazing. With two exceptions, Burnet was always the first author on the papers from his laboratory. ‘Burnet preferred to work alone, sometimes assisted by one or two graduate assistants and one or two technicians’. This does not seem like working alone to me! But Fenner tells us that ‘In consequence, many of his papers on experimental research show Burnet as the sole author and few list a co-author other than his current graduate assistant’.^12^

The exceptions are two papers with Dora Lush as the sole author. One is a description of a neutralization test for myxoma virus, the other a description of a complement-fixation test.^13,14^ As discussed under Dora Lush (1910–1943) below, her studies were undertaken by inoculating viruses onto the chorioallantoic membrane (CAM) of developing chick embryos (Box 1). This was the method of choice in Burnet's laboratory from its initial use to study ‘the virus of infectious laryngotracheitis of fowls using the developing egg technique’,^15^ through the subsequent decades of laboratory research.

Box 1. Cultivating viruses on the chorioallantoic membrane (CAM) of chicken eggs.

The highly vascularized chorioallantoic membranes of chick embryos were first used at least a hundred years ago as a site onto which to graft and grow embryonic cells and tissues.^16^

A piece of shell 0.8–1 cm is removed to create a window (windowing) and expose the underlying shell membrane. A slit in the membrane exposes the CAM onto which the tissue is grafted. The window is closed with the piece of shell and Sellotape and the egg returned to the incubator. The graft is rapidly invaded by blood vessels and begins to grow and develop. Typically, tissues are grafted into eggs that have been incubated for six or seven days. Because of the lack of an immunological response from chick embryo, grafts can be maintained until just before the embryo hatches, which is 21 days of incubation.

Subsequently, the CAM was found to allow both avian and mammalian tumours to grow and spread.^17,18^

Burnet developed a method in which viruses could be inoculated onto the CAM, where they proliferated and retained their infectivity.^15^ This became the method of choice in his laboratory for their decades-long study of viruses. An excellent video of the techniques of windowing and inoculation of viruses onto the CAM is available.^19^

Burnet produced 12 sole-authored papers over the same time (1937–1941) and, in 1940, wrote a 310-page book on *Biological Aspects of Infectious Disease.*^20^ Many of the papers are short (1–3 pages) reflecting Burnet's approach of writing up experiments as soon as the results were obtained and analyzed. However, they do include a mammoth 140-page paper on *The immunological reactions of the filterable viruses, *with Burnet, Keogh and Lush as co-authors*.*^21,22^

The role of the three assistants in the 1941 book is made explicit by Fenner in the Preface to the revised edition of 1949.^2^

In its original form it [*The Production of Antibodies*] contained in addition to a review of the literature and a *theoretical discussion for which one of us (F. M., Burnet) was wholly responsible,* an account of a good deal of experimental work in which Mavis Freeman, A. V. Jackson and Dora Rush *collaborated.* In preparing the present revision we have thought it *advisable to preserve most of this experimental material, which has not been published elsewhere in its original form.* (p. v, emphases mine)

So, several things are clear. Burnet wrote the 1941 book. Experimental material from the 1941 book has been retained in the revised edition because ‘it has not been published elsewhere in its original form’. This is ambiguous. The references cited in the 1949 book include three papers by Burnet and Freeman,^23–25^ two by Burnet and Lush,^26,27^ and one by Burnet, Keogh and Lush.^21^ All obviously predate the 1941 book but presumably do not represent the material that ‘has not been published elsewhere in its original form’. Perhaps this is nit-picking, but it is hard to find references to the co-authors of the original research in the 1949 edition.

Further enlightening is how Fenner recalls how he came to ‘co-author’ the 1949 revised edition.

Early in 1948, after he had an opportunity to evaluate my writing, Burnet asked me to collaborate with him in an article he had been asked to write for a new international journal, *Heredity.*^28^ [This is a substantial 35-page paper on the interactions between genes and the immune system, concluding that genes influence both the structure and the function of immune cells, while the immune system is influenced by both genetic variation and environmental factors.] He must have been satisfied with my performance because he then asked me to collaborate with him in producing a second edition of *The Production of Antibodies*, the first edition of which he had published in 1941. I helped chase up *some of the work done since then*, notably Medawar's studies of transplantation immunity. *Burnet was responsible for all the interpretation and speculation.*^29^ (p. 49, emphasis mine)

It is obvious that Fenner's relationship to Burnet for the revised 1949 edition was the same as the relationship of Jackson, Freeman and Lush to Burnet for the 1941 book, viz., that of a graduate assistant. But, as discussed below, their collaborations were much richer than that and included two other researchers with close ties to the WEHI, – Esmond Venner Keogh and Edward Holbrook Derrick – and the study of Q fever.

## Alan Vaughan Jackson (1912–2000)

Jackson, who graduated M.B. B.S. from The University of Melbourne in 1935, spent two years at the WEHI as one of the first fellows funded by the Federal Health Council (renamed the National Health and Medical Research Council [NHMRC] in 1937). He was appointed as assistant to Burnet to conduct research into poliomyelitis, research for which he (Jackson) received an M.D. in 1939.

Alan Jackson co-authored six papers with Burnet in 1939 and one in 1940. Dora Lush (see below) was a co-author on three of these papers (Table 1). All but one was published in the *Australian Journal of Experimental Biology and Medical Science* (the other in volume 2 of the newly established *Medical Journal of Australia*). Four are on poliomyelitis, including two on antibodies in human sera^26,30^ two a on infection of cynomolgus monkeys (the crab-eating macaque, *Macaca fascicularis*) with polio virus;^30,31^ *M. fascicularis* was something of a model primate for polio studies. Another was on herpes B (which affects primates) and pseudorabies.^32,33^

**Table 1.** Shared authorship of 25 papers from Burnet’s Laboratory between 1937 and 1941 with numbers of papers in parentheses.^a^

Burnet, Freeman, Jackson and Lush (2)	Burnet, Jackson and Lush (1)
Derrick, Burnet and Freeman (1)	Burnet, Lush and Keogh (1)
Burnet and Lush (8)	Burnet and Jackson (7)
Burnet and Freeman (5)	

^a^ A full list of these papers is included in Fenner's memoir.^10^

Jackson spent 1940 to 1945 in the Australian Army Medical Corps (retiring as Major). He ran a clinical pathology laboratory and undertook research and autopsies, largely in the tropical climate of New Guinea. He and Frank Fenner were in the same unit and collaborated in research on *Salmonella*-induced enteric fever in 50 Australian soldiers, a study that was published in the first volume of the *Medical Journal of Australia*.^34^ Fenner, who received an MBE for his research during WW-II, went on to a significant and distinguished career resulting in the elimination of smallpox s in humans and of haemorrhagic disease virus (RHDV) in rabbits (*Oryctolagus cuniculus*), for which also see Dora Lush below.

In April 1940 Alan Jackson became engaged to Edna Mavis Swan (1913–2000), one of the bright, intelligent science graduates from Melbourne (B.Sc., 1936). In November 1941 Mavis enlisted in the AAMC, working as a microbiologist and supervisor of blood transfusions at the Heidelberg Military Hospital in Melbourne. With Alan on home leave, they married in March 1942. She was discharged a month later and set out on a career that included pathology, cytology, and fund raising.

After the war ended Alan Jackson received a Nuffield Scholarship to study pathology in London, a plan that was cut short by his appointment in 1945 as Director of Pathology at the Alfred Hospital in Melbourne, a position he held for 32 years until his retirement in 1977.^35^ Mavis worked in the Pathology Department for 15 years (1962 to 1977), where she established and ran the cytology unit. Best known for her fundraising at Melbourne University, especially for the residential college International House, she was appointed MBE in 1968.^36,37^

## Mavis Louisa Freeman (1907–1992)

The second collaborator listed on the title page of the 1941 monograph, Mavis Freeman was born in Ballarat, Victoria, a city whose very existence goes back to the discovery of gold in 1851 and the Victoria Gold Rush. With a population in 1858 of 60,000 (mostly male miners) ‘The Golden City’ endured the end of gold mining and thrived to become, what is now, the third largest inland city in Australia.

A very bright girl, Mavis was Dux (from the Latin *ducere* ‘to lead’, ‘top girl’) of Forms III and IV of her Girls School and Grammar school. A prestigious scholarship took her to the University of Melbourne where in 1928 (aged 21) she earned a B.Sc. with distinction. Equipped with both a biochemistry and bacteriology background, Mavis was appointed as a research fellow at the WEHI, initially working on snake venoms and the nature and denaturation of proteins. She remained there for 12 years (1928–1940).

The award of a Victorian Women Graduates’ Association Travelling Scholarship in 1934 allowed Mavis to travel to the Lister Institute in London, returning to the WEHI in 1939 where she and Burnet began their ground-breaking studies identifying the microbe responsible for Q fever. Because Freeman, Lush (see below), Burnet and a physician, Edward Holbrook Derrick were closely and collectively involved in the studies on Q fever I have given the fever a separate section below. Just to note at this stage that Freeman was co-author on eight papers between 1937 and 1941, indicative of a remarkably productive period of research from a remarkable team.^33^

With the outbreak of WW-II, Freeman was commissioned as a lieutenant and appointed as pathology assistant to the Second Australian Hospital of the Australian Army Medical Corps, stationed in Palestine. She is said to have been the only woman to serve overseas who was neither a nurse nor a masseuse. Her superior, Esmond (Bill) Keogh (whose research and career is discussed below) was then the Director of Hygiene and Pathology for the Australian Army. She knew Keogh from the time he had spent at the WEHI learning how to inoculate viruses onto CAMs. With her joint biochemical and bacteriological background, Mavis undertook research into safe methods for blood transfusion in malarial regions, resulting in dramatic reductions in cases of malaria among troops in the Pacific. She ended WW-II as a Captain in the Australian Army Medical Corps. Freeman returned to the WEHI in 1946 but resigned in 1948 to move to Adelaide to work at the Institute of Medical and Veterinary Research.^38^

## Esmond Venner (Bill) Keogh (1895–1970)

Esmond Venner (Bill) Keogh was ‘Es’ to family and close friends, ‘Bill’ to all others. With combined skills as a physician, pathologist and medical researcher Keogh is credited with research and application of technology that saved thousands of lives of individuals with TB, polio and cancer after the end of WW-II.^39,40^

Keogh's medical education at the University of Melbourne was interrupted when he enlisted (underage) in the Australian Imperial Force, working as an ambulance officer at Gallipoli and in a machine gun company on the Western Front, where he was awarded a distinguished conduct medal for gallantry. He spent 1919 to 1922 unsuccessfully trying to farm with his father in East Gippsland. He went back to medicine, qualified in 1927, and joined the Commonwealth Serum Laboratory (CSL) which had been established in Melbourne in 1916. Over time the CSL became the major producer of medical products such as plasma, vaccines, antivenoms and reagents for cell cultures. Privatized in 1994, it was listed with the Australian Securities Exchange. It is now a global biotechnology company (CSL Ltd).^41^

With little training, CSL medical officers such as Keogh were sent all over the country, investigating TB, anthrax and other disease outbreaks. In 1935, Keogh established an independent research unit at the CSL. Burnet and Dora Lush from the WEHI taught him how to inoculate viruses onto CAMs (Box 1), a collaboration that resulted in 18 jointly authored papers between 1936 and 1941.^33^ These range from a mammoth 140-page paper on *The immunological reactions of the filterable viruses,*^21^ and a diminutive two-page paper on the virus responsible for the Victorian and Tasmanian epidemics of poliomyelitis in 1937.^42^

As soon as WW-II was announced in 1939 Bill joined the Second Australian Imperial Force (as a Major) to serve as pathologist in the Middle East. His assistant was Lieutenant Mavis Freeman (*ibid*). Treating malaria was a major occupation.^43^ Keogh resigned his position as Director of Hygiene, Entomology and Pathology in April 1945, to move (in May) to Washington, DC to become the Assistant Director General of Medical Services (ADGMS) in the Australian Military Mission to the United States, based in Washington, DC.

A year later, Keogh was transferred to the Reserve Officers with the rank of lieutenant colonel and the honorary rank of colonel, returning to the Commonwealth Serum Laboratories as Deputy Director of Research. Over the next 20 years Keogh was extraordinarily effective in (i) supporting those suffering from TB, (ii) bringing the Salk polio vaccine to Australia, and (iii) obtaining funding and facilities for cancer research.

With respect to polio vaccination*,* Jonas Salk spoke to Keogh about mass producing the Salk virus in Australia. Keogh used his connections in the US to obtain the necessary equipment and expertise. The NHMRC set up a committee consisting of Keogh, Burnet and Fenner to supervise the vaccination campaign, which was launched in July 1956. The previous three years had seen 4168 cases of polio in Australia. In the 12 months after July 1956 there were 62. By 1972 there were no locally acquired cases of polio in Australia.

Keogh was homosexual with a series of partners. How he achieved what he did in his many careers, given how homophobic the medical profession and the Australian Army were, is a testament to his amazing ability to overcome.

## Q (Query) Fever

Q (query) fever is an infection of humans, domestic and farm animals caused by the bacterium and obligate intracellular pathogen *Coxiella burnetii*. When first described, it was named ‘Q’ for ‘query’ because the causative agent was unknown. This was how terms such as ‘agent’ or ‘factor’ were often used in biology until an entity had been identified. Hormonal/humoral factors that circulate in the blood stream and that would be identified as thyroxine or corticosterone are two examples.

The symptoms of Q fever were first recorded in 1935 in a slaughterhouse worker in a Brisbane abattoir by the pathologist Edward Holbrook Derrick (see below) following the outbreak of a ‘fever’ of unknown origin. Workers who handled slaughtered animals or membranes associated with birthing products were discovered to be at especially high risk of exposure. Symptoms in cows included uterine inflammation, abortions, retention of the placenta, and subsequent infertility. Symptoms in humans – fevers, chills, extreme sweating, headaches, muscle and joint pains and extreme fatigue – were found to appear some two-to-three weeks after exposure. Derrick described the disease as having some of the clinical symptoms of typhus and typhoid fever but lacking others. He named it Q fever.^44^ Derrick, who was Director of the Laboratory of Microbiology and Pathology of the Queensland Health Department stationed in Brisbane (see below), was asked to investigate the nature of the disease. He thought it was probably a virus and sent organs from an infected guinea pig to Frank Burnet and Mavis Freeman at the WEHI. Freeman had been at the Institute since 1928 and was a very accomplished bacteriologist. Freeman was a co-author of seven papers on Q fever between 1937 and 1941.^33^

Burnet and Freeman tested suspensions of the infected cells in various animals and on the CAM. As mice showed the best results – appearance of *Rickettsia* in their enlarged spleens – they concentrated on mice as the test species. From the sera provided by Derrick, Freeman was able to prepare a stable rickettsial suspension from infected mouse spleens and to develop what has been described as ‘a “neat though somewhat tricky” micro-agglutination test for specific antibodies in human and lower animal sera’.^45^ (p. 90)

Burnet and Freeman^46^ inoculated cultures of viscera from minced 12-day old chick embryos with material from a culture of the bacterium. Bacterial growth began on the 4^th^ day and was maximal at the 6^th^ or 7^th^ day of culture. Because *Rickettsia* continue to grow during the second week of tissue culture, Burnet and Freeman concluded that the causative agent of Q fever almost certainly belonged to the Rickettsia group.^24^ Burnet concluded that ‘These results are regarded as indicating that the causative agent of Q fever almost certainly belongs to the Rickettsia group’.^24,25^ *Rickettsia* is a speciose and widespread genus of obligate intracellular Gram-negative bacteria. Derrick proposed the name *Rickettsia burnetii* for the causal organisms of Q fever, the specific epithet *burnetii* honouring Burnet's contributions to the initial study. The genus *Rickettsia* is in the class Alpha proteobacteria. Subsequently, *R. burnetii* was found to have physiological features of another class of bacteria, the Gamma proteobacteria and so was reclassified.

A slew of papers followed these initial studies, including infecting laboratory animals and comparisons of rickettsial strains in Australia and with a strain in Montana, discovered around the same time.^47,48^ Mavis Freeman was the lead author of a paper with Derrick and three others, testing sera for agglutination with an emulsion of *Rickettsia burnetii,* while Derrick used the agglutination test to investigate and unravel the epidemiology of Q fever in Queensland.^49,50^

Several decades later Derrick provided an insightful overview of research on *Coxiella burnetii*, while Omsland and colleagues emphasised the ability of the bacterium to grow outside its host.^51,52^ Indeed, Q fever and *Coxiella burnetii* became a model system in which to understand host-parasite interactions: ‘The papers of Derrick and of Burnet and Freeman remain models of careful investigations, critical analyses, and conclusions’^53^ (p. 129).

A vaccine (Q-VAX) was finally produced and licensed for use in Australia in 1989. In 2001, Australia introduced a national Q fever vaccination program for those in high-risk occupations such as veterinary research, farming or meat processing. Despite the availability of Q-VAX, Q fever remains a worldwide public health risk, with considerable genetic diversity worldwide.^54^ It can be deployed in an aerosol form, either alone or with other ‘agents’^55^ and so is a potential biological weapon. Indeed, in the 1950s and 1960s the bacterium was produced, tested and stockpiled in the US to an amount of over 5000 gallons. Now that the genome has been sequenced^56^ it is potentially even more dangerous. The Centre for Disease Control currently ranks *Coxiella burnetii* as a ‘category B bioterrorism agent’.

### Edward Holbrook Derrick (1898–1976)

Derrick was born in Victoria two years before the end of the 20th century.^45,57^ He graduated in Medicine (MB, BS) from the University of Melbourne in 1920 and was awarded the Sir John Grice Scholarship in Cancer Research at the WEHI. The scholarship provided a stipend, residence in hospital, experience assisting at autopsies and histological research on tumours of the kidneys and suprarenal glands. At the end of July 1922, Derrick obtained one of the free passages to England offered through Melbourne University by the Peninsular and Oriental (P&O) Steam Navigating Company. In London he obtained a position as pathology assistant to Hubert Turnbull (1875–1955). Turnbull was Director of the Pathological Institute at the London Hospital from 1906 to 1946, where training in post-mortems and histology was second to none. Derrick passed the primary FRCS examination in December 1923 ready to return to Australia.

An event that dominated the next five years of Derrick's life occurred when he was enjoying a brief holiday in Paris before returning to Australia. He coughed up blood. Back in London, his blood was found to contain tubercle bacilli (*Mycobacterium tuberculosis*), the causative agent of tuberculosis. He had TB, which may have been contracted during one of his post-mortems in London, although he had been in contact with family members with TB in his youth.

From Derrick's arrival back in Melbourne on 13 February 1924, he spent the next five years moving all over the country from one short-term post (locum) to another, only to find himself unable to fulfil his duties. In 1929 he was offered a ‘permanent’ position as medical officer at the Irvinebank Hospital, located in a tiny tin-mining village in the foothills of the Atherton Tablelands in Northern Queensland, south-west of Cairns. Decline in mining left the hospital board unable to pay his salary and so in May 1932 he lost his post. Derrick obtained a similar position in another mining town in North Queensland, Mount Mulligan, now deserted, where he remained until early 1934 when he set up in private practice in Brisbane.

With his family Derrick remained in Brisbane for the next 42 years, but not in private practice. He was appointed director of the Laboratory of Microbiology and Pathology in the Queensland Health Department in June 1935 where he set up detailed and complete procedures based on his experience in London; they were a model for others to follow. In August of his first year as Director, Derrick was invited to investigate what was described as ‘abattoir fever’ in the Brisbane Abattoir – Q fever.

In 1946, The Queensland Institute of Medical Research was established. Derrick was appointed acting deputy-director at the first council meeting in February, deputy-director in March 1947 and Director in July 1961, the year he was made CBE. Teams at the Institute investigated arboviruses, tumour viruses, *Rickettsia*, and bacteriology, leaving Derick to concentrate on the epidemiology of asthma, a disease that was increasing in Brisbane and on which he concentrated, producing 19 papers, 13 of them after his ‘retirement’. A *Festschrift,* consisting of three short eloges by Burnet, a physician (D. W. Johnson), and R. L. Doherty was published in his honour in the *Medical Journal of Australia *in December 1967*.*^58–60^ An obituary in the November issue cited Burnet's summary that ‘to have defined and elucidated the aetiology of two worldwide infectious diseases (Q fever and Leptospirosis in cattle/ ‘swineherd's disease’ in Switzerland caused by *Leptospirosis pomona*)^45^ is something no other living scientist can claim’ (p. 732).^61^

### Dora Lush (1910–1943)

Dora Lush,^62–65^ the third collaborator on the monograph was an incredibly impressive bacteriologist as has been reemphasized recently in a memoir published in the journal, *Immunology and Cell Biology* (*ICB*) to commemorate the 100^th^ anniversary of the journal that started in 1924 as the *Australian Journal of Experimental Biology and Medical Science.*^63^ Burnet and his colleagues published most of their papers in the early issues of this journal. In 1987 the journal was renamed *Immunology &mpa#amp;and Cell Biology* and became the official journal of the Australasian and New Zealand Society for Immunology, Inc.

**Figure 2.**
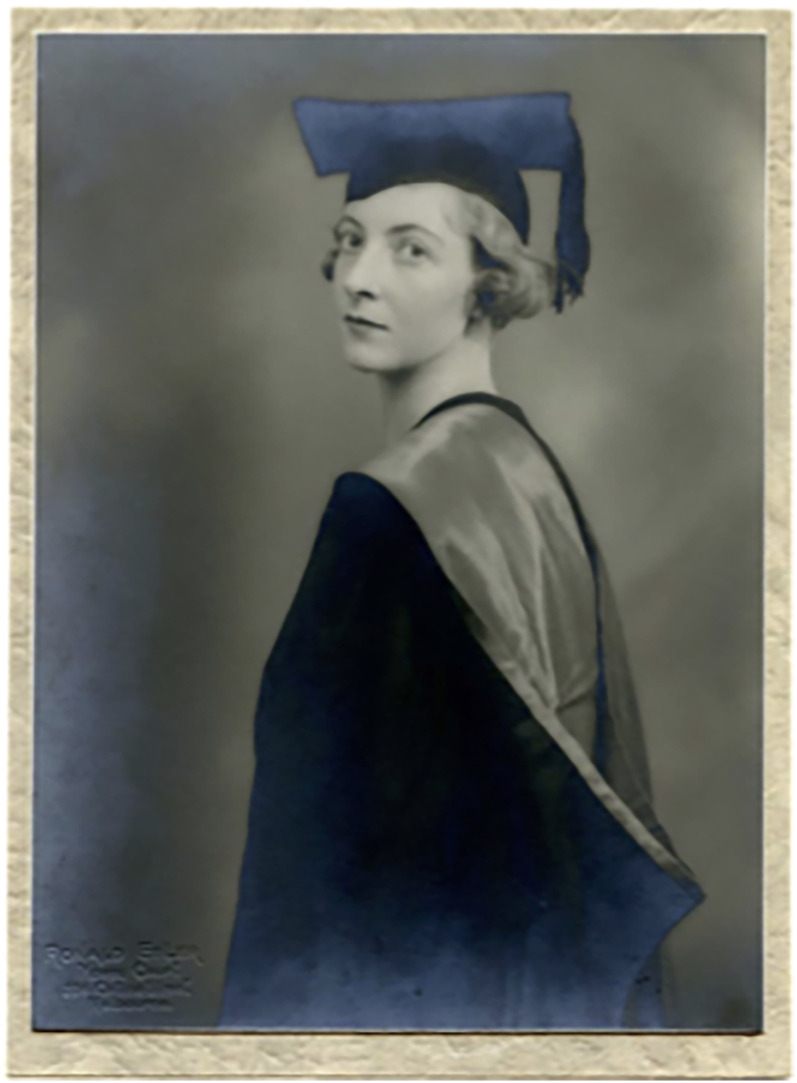
Dora Lush in her M.Sc. robes (1934) and, no doubt, 2-inch heels.

Source: https://www.wehi.edu.au/about/history/notable-scientists/dora-lush/.

Growing up in Melbourne with a supportive and extended family, at age 18 Dora took up physics and chemistry, was Dux of her school (as Mavis Freeman had been) and won an Exhibition to the University of Melbourne from which she graduated B.Sc., and M.Sc. (1932, 1934)^66^ (Figure 2). Australian newspaper records show her as a glamorous 1929 debutante, vice-captain of the women's basketball team, on the ski slopes at Mt Hotham, and attending numerous balls and social events.^67^ Tall and slim with wavy auburn hair, athletic (squash, skiing) and an excellent dancer she could have been a professional in any of these activities. Always groomed immaculately, she wore two-inch heels, even in the laboratory.

Upon graduating with her Masters, Dora was appointed as a bacteriological research fellow to work with the then assistant director of the WEHI, Frank Burnet, on infectious diseases including influenza, herpes infections^32^ and myxomatosis (see below). Within five years she was a co-author of six (and sole author of two) papers in the *Australian Journal of Experimental Biology and Medical Science.*

### Influenza, myxomatosis and scrub typhus

The isolation and identification of influenza A in 1933, renewed interest in studying a myxoma virus from the *Leporipoxvirus* genus known to produce lethal myxomatosis in rabbits. Rabbits had become a nation-wide pest in Australia by the 1930s. Although they ‘came over’ with the First Fleet, the big event was the release of 24 rabbits for sport onto one ‘property’ in NSW in 1859. Within 20 years, there were millions (rabbits not properties), by the 1920s 10 billion rabbits (Department of Agriculture and Food. https://casestudyofrabbits.weebly.com/case-study-of-rabbits.html).

Lush inoculated myxoma virus onto the CAM (Box 1) to test whether the virus would produce lesions ‘cross-species’. She assumed that failure of the virus to grow on the CAM would be because of species specificity – it was a rabbit not a chicken virus. To her surprise, subcutaneous tissue from an infected rabbit grafted onto the CAM formed foci of immune cells within only a few days. The lesions regressed within a week, but the developing chicken embryos accumulated virus in their livers, which were still fully infective when injected into rabbits.^36^ She also found that myxoma virus inoculated onto the CAM could be inactivated with fibroma antiserum.^14^

In a further study, Lush and Burnet isolated strains of virus from individuals affected in the Melbourne influenza epidemic of 1939.^68^ What appeared to be a *homogeneous* epidemic producing similar symptoms, including fever, perspiration, headaches*,* muscle and joint pain, respiratory and gastrointestinal problems, was traced to *antigenically different strains* of the virus. Their extrapolation was that: ‘it would be a natural extension of this idea to believe that in pandemic periods even wider variation might result, that in this way a reasonable explanation of the multiple waves in a pandemic as that of 1918–1919 (H1-N1 influenza pandemic) may be obtained’^68^ (p. 49).

With such experience and knowledge behind her, in 1939, when WW-II was declared, Dora Lush went, with Burnet's blessing, to pursue research on poliomyelitis at the National Institute for Medical Research (NIMR) in London. The outbreak of WW-II changed all that. For two years she worked with the team led by the Scottish born virologist (Sir) Patrick Laidlaw (1881–1941) investigating air-borne infections, first influenza, then immunization against typhus. Laidlaw was co-author on the paper reporting the isolation of the influenza A virus from humans,^69^ initiating a pivotal period in our understanding of influenza and the development of vaccines. The influenza B virus was isolated three years later,^70^ the same year that Burnet^15^ discovered that influenza viruses could be grown on the CAMS of chick embryos.

Dora returned to Melbourne in September 1942 to continue the research she had started in London to find a vaccine for the bacterial disease scrub typhus (bush typhus) that had caused such devastation among soldiers in the Pacific during WW-II with fever, headache, body aches and a rash. The bacterium, *Orientia tsutsugamushi* is spread through bites from larval mites. Scrub typhus could only be maintained by inoculations of blood from animal to animal. Lush used mice.

Lush had only been back from London for seven months when, on 27 April 1943, she pricked the index finger of her left hand while inoculating a mouse with a particularly malignant strain of scrub typhus. She died only a little over three weeks later, on May 20^th^. She was 32. Ever the scientist, from her bed in the Royal Melbourne Hospital Dora insisted that blood samples be taken at regular intervals to assist research into scrub typhus, a heroic effort that had, and still has, her hailed as a martyr to science and to the war effort. Burnet said it all: ‘It is difficult to express how grievous is her loss to the institute. She was the most outstandingly competent bacteriologist with whom I have ever worked’.^67^

**Figure 3.**
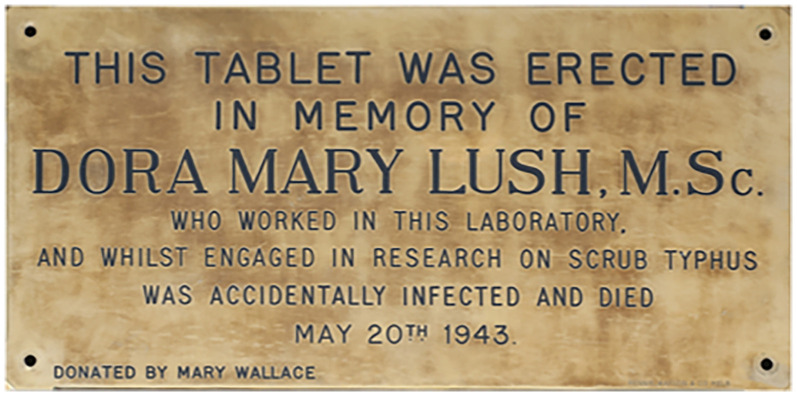
The memorial tablet to Dora Rush outside the WEHI lab where she undertook her research.

A memorial tablet hangs outside the laboratory where she worked in her last days (Figure 3). Scholarships for maths and science were established at Fintona Girls School in her name. The NHMRC established biomedical research postgraduate scholarships in her name as did her family through the annual Dora Lush Travel Fellowship at the Burnet Institute.

Source https://www.wehi.edu.au/about/history/dora-lush-career/.

## Concluding comments

So, clearly Burnet ‘wrote’ the 1941 book and the 1949 second edition, even though Fenner is credited as co-author of the second edition. That said, the role of the three collaborators – Freeman, Jackson and Lush – for the first edition was critical in providing experimental evidence regarding the production of antibodies. While Burnet set the topic on which they would work they, especially Freeman and Lush, were given substantial independence and the ability to interact and collaborate on projects. The range of topics was vast – influenza, poliomyelitis, myxomatosis, herpes, Q fever and scrub typhus. This period, 1937 to 1941, was enormously productive (25 research papers), providing evidence that was foundational in immunology, with consequences that remain today.

This analysis of the research by Freeman, Jackson and Lush illuminates how effective Burnet was in building his team and how skilled and inventive his assistants were. Although Burnet was the first author on all but two papers from his laboratory, his discussion of their experiments in *The Production of Antibodies* shows the value he placed on them and their work and provides a much richer and broader context of their pioneering research in virology and immunology.
